# Peripheral monocytes from Crohn’s disease patients retain functional responsiveness to GM-CSF during active disease

**DOI:** 10.3389/fimmu.2025.1663713

**Published:** 2025-09-23

**Authors:** Paul P. Winkel, Wendy Bergmann-Ewert, Johannes Reiner, Astrid Huth, Dana Kleimeier, Rosaely Casalegno Garduño, Ida M. Wrobel, Grazyna Domanska, Jan Däbritz, Elisa Wirthgen

**Affiliations:** ^1^ Rostock Medical School, Rostock University, Rostock, Germany; ^2^ Core Facility for Cell Sorting and Cell Analysis, Rostock University Medical Center, Rostock, Germany; ^3^ Division of Gastroenterology, Department of Medicine II, Rostock University Medical Center, Rostock, Germany; ^4^ Department of Anesthesia, Critical Care, Emergency and Pain Medicine, Greifswald University Medical Center, Greifswald, Germany; ^5^ Institute of Experimental Gene Therapy & Cancer Research, Rostock University Medical Center, Rostock, Germany; ^6^ Department of Transfusion Medicine, Rostock University Medical Center, Rostock, Germany; ^7^ Department of Immunology, Greifswald University Medical Center, Greifswald, Germany; ^8^ Department of Pediatrics, Greifswald University Medical Center, Greifswald, Germany; ^9^ Institute for Clinical Research and Systems Medicine, HMU Health and Medical University, Potsdam, Germany; ^10^ Department of Pediatrics, Klinikum Westbrandenburg, Potsdam, Germany; ^11^ Department of Pediatrics, Rostock University Medical Center, Rostock, Germany; ^12^ University of Applied Sciences Zittau/Görlitz, Faculty of Natural and Environmental Sciences, Zittau, Germany

**Keywords:** Crohn’s disease, monocytes, cellular therapy, granulocyte-macrophage colony-stimulating factor, cytokine, innate immunity

## Abstract

**Introduction:**

Crohn’s disease (CD) is a relapsing inflammatory disease that is currently not curable. Despite the availability of anti-inflammatory treatments, 30–60% of patients develop resistance, necessitating the need for novel therapeutic approaches. Data from colitis models in mice indicate that granulocytemacrophage colony-stimulating factor (GM-CSF)-activated monocytes (GMaMs) may serve as a promising cellular therapy. However, it remains unclear whether inflammatory mediators, medication, or intrinsic defects impair the functionality of monocytes in active CD (aCD), potentially affecting their therapeutic efficacy.

**Methods:**

Monocytes from 8 aCD patients and healthy donors (HDs) were activated in vitro with GM-CSF, and their migratory capacity, adherence, metabolic activity, surface marker expression, and cytokine release were assessed. Cells were stimulated with lipopolysaccharides (LPS) to evaluate their inflammatory response.

**Results:**

The GMaM phenotype was characterized by increased metabolic activity, enhanced production of inflammatory cytokines, stronger adhesion, and remodeling of surface receptors involved in T-cell activation, compared to naïve monocytes. These features were largely comparable between aCD patients and HDs. LPS stimulation of GMaMs from both groups resulted in a significant production of pro-inflammatory and chemotactic cytokines, particularly interleukin (IL)-8 and monocyte chemotactic protein 1. Both are crucial recruiters of neutrophils and monocytes. Notably, monocytes from aCD patients showed an increased IL-10 response to GM-CSF, while the LPS-induced tumor necrosis factor-alpha and interferon-gamma release were reduced compared to HDs.

**Discussion:**

Peripheral monocytes from aCD patients retain functional responsiveness to GM-CSF activation, displaying preserved migratory, adhesive, metabolic, and cytokine responses. Differences in cytokine production by aCD monocytes may reflect disease-specific regulation, but do not appear to limit their suitability as cellular therapeutics.

## 1 Introduction

Crohn’s disease (CD) is a chronic relapsing inflammatory bowel disease (IBD), primarily affecting the gastrointestinal tract, with potential extraintestinal manifestations (1). Due to its rising incidence, prevalence and its associated systemic symptoms, CD concerns an increasingly diverse group of clinicians (2). Current treatment includes the use of medication, dietary adjustments, and surgical procedures. However, these are not curative (3). Medications in IBD are designed to systemically suppress the immune system using immunosuppressants (e.g., corticosteroids), immunomodulators (e.g., thiopurines), biologics (e.g., anti-tumor necrosis factor alpha, TNF-α) and/or small molecule drugs (e.g., Janus kinase inhibitors), all of which have potential systemic side effects such as an increased risk of infection and the development of malignancies (4). In addition, roughly 30% of patients fail to respond to anti-TNF-α treatment after one year and 60 to 70% after 3 years of treatment (5), requiring the development of new approaches to the treatment and monitoring of IBD.

One novel approach is to utilize the immunomodulatory effects of endogenous cytokines, such as granulocyte-macrophage colony-stimulating factor (GM-CSF), to activate monocytes *ex vivo*. GM-CSF is not only a hematopoietic growth factor for bone marrow progenitor cells but also mediates various pleiotropic effects on mature myeloid cells, such as monocytes and macrophages (6). In chronic inflammation, peripheral monocytes have been found to be potentially responsive to GM-CSF, making them interesting candidates for regulating inflammation. GM-CSF activation led to amplified cytokine production, primarily of pro-inflammatory cytokines (IL-6, IL-23, and CCL17); however, studies suggest that an additional mediator, such as LPS, may be necessary. Additionally, GM-CSF led to an upregulation of major histocompatibility complex (MHC) class II molecule, thereby enhancing antigen presentation by monocytes (6, 7). Based on data derived from mouse models of experimentally induced colitis, we hypothesize that GM-CSF-activated monocytes (GMaMs) represent a specific population of monocytes with protective and therapeutic effects on intestinal inflammation. Mice were found to have a decreased colitis score, decreased gut shortening, and less weight loss after treatment with GMaMs (7–10). Based on the observation that no effect was observed in T-cell-deficient mice, it was concluded that the beneficial effects of GMaMs are T-cell-dependent, including the proliferation and differentiation of anti-inflammatory and regulatory T-cell subsets in the intestine. Further studies have shown that *ex vivo* GM-CSF stimulation of human monocytes induces an immunoregulatory phenotype (6) characterized by altered functional properties, including increased cell adhesion, migration, and chemotaxis, which may facilitate their infiltration into inflamed tissue. It’s noteworthy that the concept of GMaMs is based on immune cell stimulation rather than suppression, representing a novel treatment concept compared to currently used immunosuppressants. Therefore, the pro-inflammatory properties of GMaMs might be advantageous in aCD, where defects in innate immunity contribute to impaired bacterial clearance and delayed resolution of inflammation (11–13). By producing chemotactic cytokines such as IL-8 and MCP-1, GMaMs may facilitate the recruitment of neutrophils and monocytes to the site of inflammation, enhancing microbial clearance and debris removal. In addition, through secretion of T-cell–attracting chemokines such as CCL17 and CCL22 and the upregulation of co-stimulatory molecules including CD86 and HLA-DR, GMaMs can promote the recruitment and activation of CD4^+^ T-cells with regulatory or anti-inflammatory functions (7, 9).

Interestingly, previous studies of peripheral blood monocytes from CD patients in clinical remission have expressed similar GM-CSF-induced effects *ex vivo* when compared to those from healthy donors. These findings suggest that there is no general disease-induced intrinsic defect in monocytes that could prevent an adequate GM-CSF response (11). Although studies in patients in remission were promising, it remains unclear whether inflammatory mediators, medication, or intrinsic defects might impair the functionality of peripheral monocytes in patients with active CD (aCD), limiting their intended therapeutic effect (11). We hypothesized that, despite active inflammation, peripheral monocytes from aCD patients retain the capacity to respond to GM-CSF activation in a manner comparable to that of healthy donors (HDs) and that this response is functionally sufficient to support their intended therapeutic application as GMaMs.

To test our hypothesis, classical monocytes of aCD patients were isolated and activated *ex vivo* with GM-CSF. Their response was characterized by the measurement of cytokine secretion and surface protein expression related to homing, migration, and intercellular communication. The resulting GMaMs were tested in functional assays for adherence, migratory capacity, metabolic activity, and cytokine production. Moreover, GMaMs were stimulated with lipopolysaccharides (LPS) as an experimental model for the response to potential intestinal bacterial stimuli. To identify the inflammatory profile of GMaMs following LPS stimulation, we evaluated markers of both pro- and anti-inflammatory responses. We included measurements of Tryptophan (TRP) and Kynurenine (KYN) as markers of an anti-inflammatory pathway relevant to monocytes’ LPS response (14). The degradation of TRP by Indoleamine 2,3-dioxygenase (IDO) along the KYN pathway plays a crucial role in the regulation of the immune response, notably as a counter-regulatory mechanism in the context of inflammation and immune metabolism (15). It has been shown that IDO expression in dendritic cells or macrophages suppresses T-cells by depriving TRP (16). Additionally, it has been shown that KYN inhibits T-cell-mediated inflammation by suppressing T-cell proliferation (17). As a control, all tests were also performed with monocytes from HDs to account for effects related to active disease in the monocytes of aCD patients.

**Figure 1:**
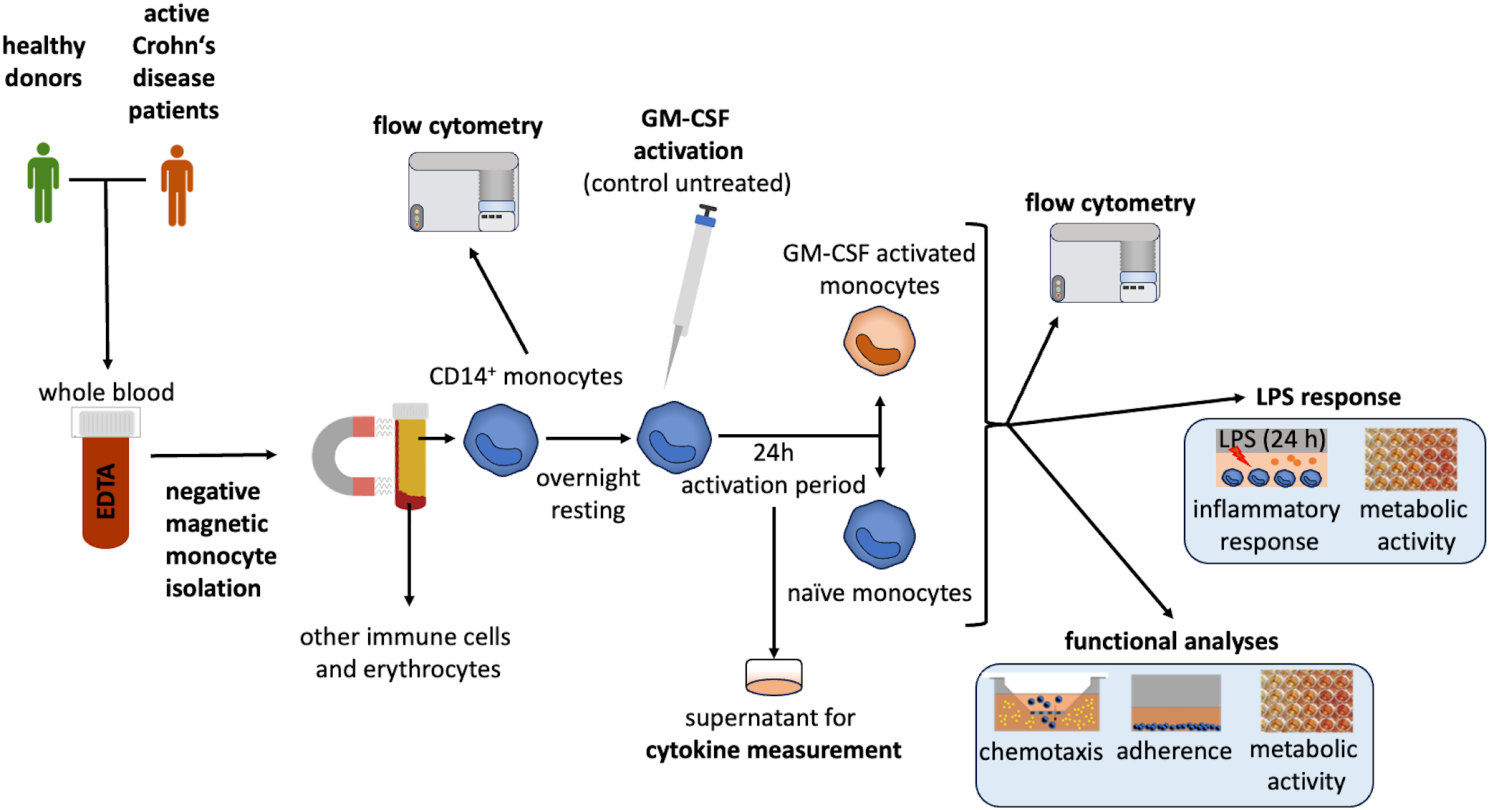
Experimental design. CD14^+^ monocytes were isolated from whole blood using negative selection, as previously described (20). Cell size and yield were analyzed directly after isolation. The isolated monocytes were diluted to a concentration of 1 x 10^6^ cells/mL and seeded in a cell-repellent multiwell plate (Greiner Bio-One, Frickenhausen, Germany). Monocytes rested overnight. Cells were then activated by GM-CSF (to polarize them into GMaMs) or remained unstimulated (naïve). Monocytes were cultivated for 24h. After cultivation (44h after isolation), cells were harvested and their number, size and viability were evaluated using the CASY cell counter (60). Afterward, GMaMs and naïve monocytes were examined for their functionality (migratory capacity, adherence, metabolic activity, GM-CSF-induced cytokine production). Furthermore, the response of GMaMs and naïve cells to LPS was characterized by quantifying pro- and anti-inflammatory mediators (cytokines and Tryptophan (TRP) metabolites) and metabolic activity. Cell culture supernatants were collected after both GM-CSF activation and LPS stimulation and stored at -20°C for cytokine and metabolite analysis. For the analysis of the monocytes using flow cytometry, a portion of the cells was cryopreserved immediately after isolation from peripheral blood and after incubation with or without GM-CSF. CD, Cluster of differentiation; EDTA, Ethylenediaminetetraacetic acid; GM-CSF, granulocyte-macrophage colony-stimulating factor; LPS, Lipopolysaccharide.

The experimental design used in this study is illustrated in **Figure 1**.

## 2 Materials and methods

### 2.1 Patients and controls

Monocytes were isolated from whole blood of adults after obtaining written consent for scientific use (approval numbers: A2016–0046 and A2021-0229, Rostock University Ethics Committee). Patient samples were provided by the Department of Gastroenterology at Rostock University Medical Center, Germany (n = 12). Patients were included in the study based on the physician’s assessment, which indicated either an acute relapse of existing CD or the initial diagnosis of active CD. In addition, patients had to meet the criteria of the Crohn’s Disease Activity Index (CDAI) (18), defining moderate and acute disease activity with a score of at least 221. One patient was excluded from the study because their CDAI was less than 220. Moreover, two additional patients were excluded as their CD diagnosis was revised to ulcerative colitis following further diagnostic tests. One more patient was excluded because the diagnosis of CD was changed to bacterial gastroenteritis, resulting in a final number of 8 included patients with aCD. To further characterize the disease activity of the patients, laboratory parameters associated with active inflammation were included, such as leukocytes, hemoglobin (Hb), C-reactive protein (CRP), and fecal calprotectin, which is used to classify the severity of gastrointestinal inflammation. The patient’s characteristics, including disease activity location, inflammation parameters, CDAI, and medication, are summarized in **Table 1**.

**Table 1:** Patient characteristics.

Crohn’s disease patients with active disease (n = 8)								
Gender	F	F	F	M	F	M	M	F
Age (years)	37	33	28	24	38	19	21	54
Disease duration (years)	7	<1	11	5	7	<1	3	<1
Treatment								
None	+	–	–	–	–	+	+	+
Topical corticosteroid	–	–	+	+	–	–	–	–
Systemic corticosteroid	–	+	–	+	–	–	–	–
IL-12/IL-23 antibody	–	–	–	+	+	–	–	–
α4β7-Integrin inhibitor	–	–	+	–	–	–	–	–
Disease location								
Upper GI tract	–	–	–	–	–	–	–	+
Ileum	–	+	+	+	–	+	+	–
Terminal ileum	+	–	–	–	+	–	–	+
Colon	–	+	+	+	–	+	–	+
Disease behavior								
Extraintestinal manifestations	+	+	–	+	+	–	+	–
Corticosteroid refractory	–	+	–	–	+	–	–	–
Structuring disease	–	–	–	+	–	–	+	–
Clinical measurements								
CDAI (score)	498	234	259	309	301	419	251	252
Leukocytes (10^9^/L)	13.2	15.0	8.8	8.8	7.1	6.8	6.5	7.5
Hemoglobin (mmol/L)	8.2	9.1	9.1	8.8	8.3	6.9	8.8	7.5
C-reactive protein (mg/L)	104	5.3	13.1	52.8	4.8	108	6.7	3.3
Calprotectin (mg/kg)	348	36	<22	167	n/a	4188	357	n/a

M, male; F, female; CDAI, Crohn’s Disease Activity Index; GI, gastrointestinal; IL, interleukin; TNF, tumor necrosis factor; +, characteristic applies to patient; –, characteristic does not apply to patient; n/a, data not available.

Control blood samples were obtained from HDs, which were provided by the Department of Transfusion Medicine at Rostock University Medical Center, Germany (n = 10). HDs were included based on their ability to donate blood and not fulfilling exclusion criteria, such as having an acute infection, an infection within the past 30 days, a chronic inflammatory disease, and/or taking immunosuppressing medication.

### 2.2 Isolation, cultivation conditions, and stimulation of monocytes with GM-CSF or LPS

Peripheral blood monocytes were isolated by negative selection from whole blood drawn from aCD patients and HDs using the EasySep™ Direct Human Monocyte Isolation Kits (STEMCELL, Vancouver, Canada). Accordingly, classic monocytes were obtained (CD14^+^/CD16^-^) with preserved functionality, as shown in preliminary experiments (19). Based on the measured cell count, the cell suspension was adjusted to 1 x 10^6^ viable cells/mL by adding RPMI 1640 cell culture medium (specification: very low endotoxin), which was supplemented with 10% fetal bovine serum (FBS), 100 U/mL penicillin, 2 mM stable glutamine and 0.1 mg/mL streptomycin (all: PAN-Biotech, Aidenbach, Germany). Cells were seeded in a cell-repellent plate (Greiner Bio-One, Frickenhausen, Germany) and incubated overnight at 37°C, 5% CO_2_, and 95% relative humidity (resting phase). These incubation conditions were used for all experiments. Activation of peripheral blood monocytes was achieved by adding GM-CSF (10 ng/mL, PAN-Biotech, Aidenbach, Germany), while naïve monocytes were left untreated as a control. Monocytes were further cultivated for 24h as previously described (20). To assess the monocytes’ function and reaction to bacterial stimuli, we seeded the harvested activated and naïve monocytes into a 96-well plate (100,000 viable cells/well, in triplicate) and stimulated them with or without 50 ng/mL LPS (*Escherichia coli* O111:B4, Sigma-Aldrich, Taufkirchen, Germany) for 24h as previously described (19).

### 2.3 Yield, cell size and viability measurement

To assess monocyte yield, cell size and viability after cell isolation and culture, the cell suspension was analyzed using the CASY Cell Counter (OLS OMNI Life Sciences, Bremen, Germany) according to the manufacturer’s instructions and previously described in detail (20). To monitor the monocytes’ cell size (changes in their diameter), we used the mean cell diameter in µm (20).

### 2.4 Metabolic activity, adherence, and migratory capacity

Functional analyses were performed as published (20), with some modifications. In brief, the metabolic activity was assessed using the water-soluble tetrazolium salt (WST-1) (Roche, Basel, Switzerland) assay. After one hour of incubation, the optical density was measured at 440/650 nm using the Infinite M200 plate reader (TECAN, Männedorf, Switzerland). To measure the adherence of the monocytes, cells were activated and seeded in triplicate and incubated for 2.5h. The supernatants were discarded, and the wells were washed with phosphate-buffered saline (PBS). Adherent cells were detached using Accutase (PAN-Biotech, Aidenbach, Germany) according to the manufacturer’s instructions and measured using the CASY cell counter (OLS OMNI Life Sciences, Bremen, Germany).

To evaluate migratory capacity, monocyte migration through 5-µm pores of a transwell insert suspended in a well following a monocyte chemoattractant protein-1 (MCP-1) gradient (25 ng/mL, R&D Systems, Minneapolis, MN, USA) was analyzed. Cells were seeded in the inserts and then incubated for 4h. Afterwards, cell culture supernatants in the wells containing non-adherent monocytes were collected. Adherent cells from the bottom of the wells were detached using Accutase (PAN-Biotech, Aidenbach, Germany). To also account for monocytes that adhered to the bottom of the transwell insert after migrating through the pores rather than accumulating in the cell culture supernatant, the assay was refined. We therefore additionally measured these cells by detaching the monocytes using Accutase (PAN-Biotech, Aidenbach, Germany). Detached cells were combined with their respective previously collected cell culture supernatant. Cells were counted using the CASY cell counter (OLS OMNI Life Sciences, Bremen, Germany). Total migration was calculated by adding the number of cells adherent to the bottom of the insert and the number of cells in the bottom chamber.

### 2.5 Quantification of cytokine release

Cytokines were measured in supernatants collected after GM-CSF activation or LPS stimulation, as per the experimental design (**Figure 1**). Aliquots were stored at -20°C until measurement. Interleukin (IL)-1β, interferon (IFN)-α2, IFN-γ, TNF-α, MCP-1, IL-6, IL-8, IL-10, IL-12p70, IL-17A, IL-18, IL-23, and IL-33 were measured in duplicates by flow cytometry (Cytek Aurora, Cytek Biosciences, California, USA) using the LegendPlex™ Human Inflammation Panel (BioLegend, San Diego, CA, USA). As MCP-1, IL-6, and IL-8 exceeded detection limits in the LegendPlex™ assay, samples were further measured these cytokines with ELISA kits (DuoSet^®^, R&D Systems, Minneapolis, MN, USA) according to the manufacturer’s instructions.

In addition to pro- and anti-inflammatory markers, we calculated the TNF-α/IL-10 and the IL-18/IL-10 ratios to assess the balance between pro- and anti-inflammatory responses of GMaMs and naïve monocytes. The TNF-α/IL-10 ratio has previously been described as an indicator of a balanced immune response, where higher ratios (TNF-α > IL-10) were recognized as indicative of pro-inflammatory immune activity. In comparison, lower ratios (TNF-α < IL-10) indicated a more balanced and anti-inflammatory response (21–23). The IL-18/IL-10 ratio was previously seen as an indicator of the inflammatory state, where a higher ratio suggests a more pronounced pro-inflammatory response and a reduced ratio indicates a stronger anti-inflammatory response. This measure has been proposed as a biomarker to assess disease severity and predict outcomes in various conditions (24–26).

### 2.6 Quantification of tryptophan, kynurenine and quinolinic acid

Tryptophan (TRP), kynurenine (KYN) and quinolinic acid (QUIN) were measured in cell culture supernatants of LPS-stimulated GMaMs or naïve cells using liquid chromatography-tandem mass spectrometry (LC-MS/MS). Samples were stored in aliquots at -20°C until measurement. The methodology was adapted to a newer mass spectrometer following previously published data (27). After the separation of the sample by LC (Agilent Technologies, Santa Clara, USA), it was transferred to the tandem MS (AB Sciex 5500 QTrapTM, AB Sciex, Darmstadt, Germany). The first analyzer selected ions according to their mass-to-charge ratio. The selected molecules were then transferred to the collision chamber, where they were fragmented by collision with nitrogen. The resulting fragmented ions were selected in a second analyzer based on their mass-to-charge ratio and then detected. Calibration curves were fitted using linear least-squares regression and correlated with the concentrations of d6-Kyn (Cambridge Isotope Laboratories, St. Louis, MO, USA) and d5-TRP (Sigma-Aldrich, St. Louis, MO, USA) as internal standards. Mass transitions, declustering potential (DP), and collision energy (CE) for each measured molecule are indicated in **Supplementary Table 1**. The within-run and between-run precision was calculated using quality controls of RPMI 1640 cell culture medium (10% FBS) spiked with TRP and KYN, as shown in **Supplementary Table 2**. As an indicator for activation of the KYN pathway, the ratio of KYN and TRP was calculated (KYN*100/TRP) in all samples (28).

In addition to TRP and KYN quantification, we assessed QUIN as a downstream metabolite of the TRP/KYN pathway and a precursor in the *de novo* synthesis of nicotinamide adenine dinucleotide (NAD^+^) (29).

### 2.7 Flow cytometry analysis

Before analysis, monocytes were cryopreserved both immediately after isolation and after 24h of incubation with or without GM-CSF. The cell suspension was centrifuged for 5min at 300g and the cell pellet was resuspended in animal-component-free, defined cryopreservation medium with 10% dimethyl sulfoxide solution (DMSO) (CryoStor^®^, STEMCELL, Vancouver, Canada) and cryopreserved using slow temperature lowering method (Mr. Frosty™ vial holder, Thermo Fisher Scientific, Waltham, MS, USA) to ensure cells´ fitness (30). Cells were thawed as standardized. After centrifugation at 300g (5min), cells were incubated in PBS with Zombie Viability Dye NIR (BioLegend, San Diego, CA, USA). The following antibody-fluorochrome conjugates were then added: CD14 (PerCP-Vio700), CD16 (VioBlue), CD11b (PE-Vio770), CCR2 (CD192, VioBright FITC), CX3CR1 (PE), CD64 (PE-Vio615), HLA-DR (VioGreen), and CD54 (ICAM-1, APC), all from Miltenyi Biotec (Bergisch Gladbach, Germany), as well as CD86 (Super Bright 645, Thermo Fisher Scientific, Waltham, MA, USA). Antibodies were used at concentrations recommended by the manufacturers. The functions of these markers are presented in **Supplementary Table 3**. Monocytes were washed, resuspended in cell staining buffer (BioLegend, San Diego, CA, USA), and acquired in a Cytek^®^Aurora (Cytek Biosciences, Fremont, CA, USA). Flow cytometry analysis was performed using FlowJo software (Becton Dickinson and Company (BD), Franklin Lakes, NJ, USA).

To avoid misidentifying spillover from one fluorochrome into another channel of interest as a true positive signal, we employed the fluorescence-minus-one (FMO) method as previously described in (31). Briefly, each FMO control omits one marker while including all others in the panel, allowing for the identification of signal spread and more accurate gating. FMO controls were applied to all above mentioned surface markers used in this study. This is exemplarily shown in **Supplementary Figure 1**.

**Figure 2:**
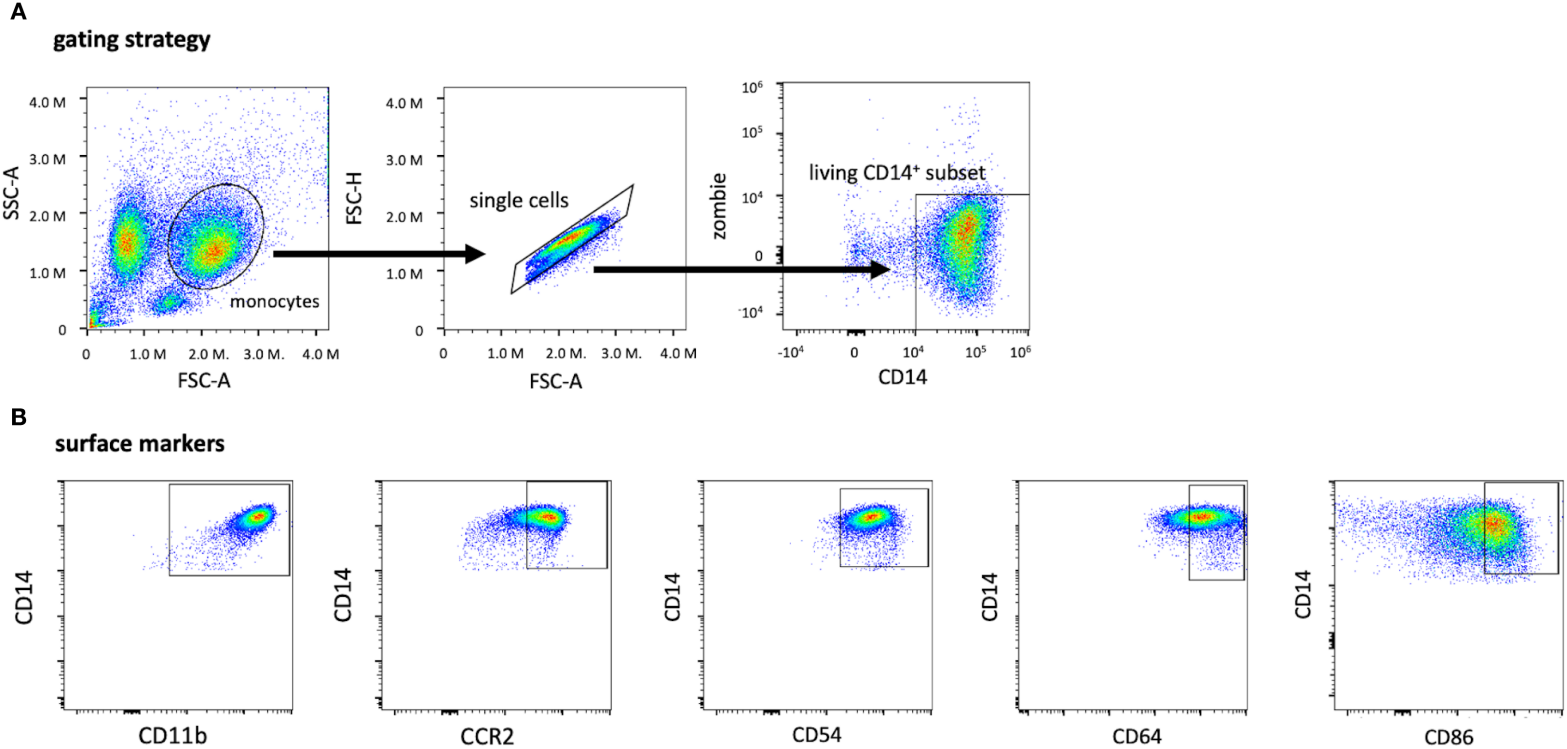
Gating strategy for identifying living CD14**^+^** monocytes and subsets of CD14**^+^** monocytes expressing other surface markers. The monocyte population was first identified by applying the singlet gate and gating on the living CD14^+^ Zombie-gate **(A)**. Based on the living CD14^+^ subset, these graphs depict the expression of the surface markers CD11b, CCR2 (CD192), CD54, CD64, and CD86 after monocyte isolation **(B)**. The threshold for CD14^+^, Zombie- cells and the surface markers were evaluated by using the FMO method with the full stain containing all selected colors on the panel. CD, cluster of differentiation; FMO, Fluorescence minus one; FSC-A, forward scatter area; SSC-A, sideward scatter area.

To characterize the expression of surface markers on live isolated monocytes, cells were gated on Zombie Viability Dye NIR (BioLegend, San Diego, CA, USA) negative and CD14 positive populations (32) (**Figure 2**).

### 2.8 Statistics

Statistical analyses were performed using GraphPad Prism version 10.2.3 (GraphPad Software Inc., San Diego, CA, USA) and “R” statistics software version 3.6.2 (R Core-Team) (33). Univariate analyses and pairwise comparisons between two groups were performed using the Student’s t-test (for parametric distributions) or the Wilcoxon rank test (for non-parametric distributions). GraphPad Prism does not provide a means to perform an ANOVA or comparisons for multivariate models with response variables from the closed [0, 1] interval (e.g., adherence, migratory capacity, proportion of surface markers). Therefore, such analyses were performed using generalized linear mixed models assuming a binomial distribution and using the logit link function. The analysis was performed using R statistics software and the emmeans package (33) for multiple comparisons of least-squares mean estimates. Due to the left-censored distribution of cytokine data, the measured concentrations were logarithmized (log10) in R and then applied to a linear model, taking repeated measurements into account using the random statement. To assess the effect of GM-CSF stimulation on cytokine secretion, adherence, metabolic activity and migratory capacity of monocytes, we applied ANOVA models with stimulant (GM-CSF, w/o), study group (aCD, HD) and their interaction (stimulant*study group) as fixed effects and donor ID as a random effect. For evaluation of the LPS response of GMaMs, ANOVA consisted of the fixed effects stimulant (GM-CSF, w/o), treatment (LPS, w/o), study group (aCD, HD), their multiple interactions (study group*stimulant, study group*treatment, stimulant*treatment*study group) and the donor ID as a random effect. The results of flow cytometry were analyzed directly after isolation (A) and after activation with (B) or without (C) GM-CSF for each study group. Accordingly, fixed effects included condition (A, B, C), study group (aCD, HD) and their interaction condition*study group. Multiple pairwise comparisons of the least square means were corrected using the Tukey multiple comparisons test. Significant differences were considered as p < 0.05 (*), p < 0.01 (**), and p < 0.001 (***). Data are presented as single points for each value measured as recommended for datasets with small *N* (34). Furthermore, means + standard deviation (SD) were presented as bars.

## 3 Results

### 3.1 Increases in monocyte size and cytokine secretion following GM-CSF activation in aCD and HD

**Figure 3:**
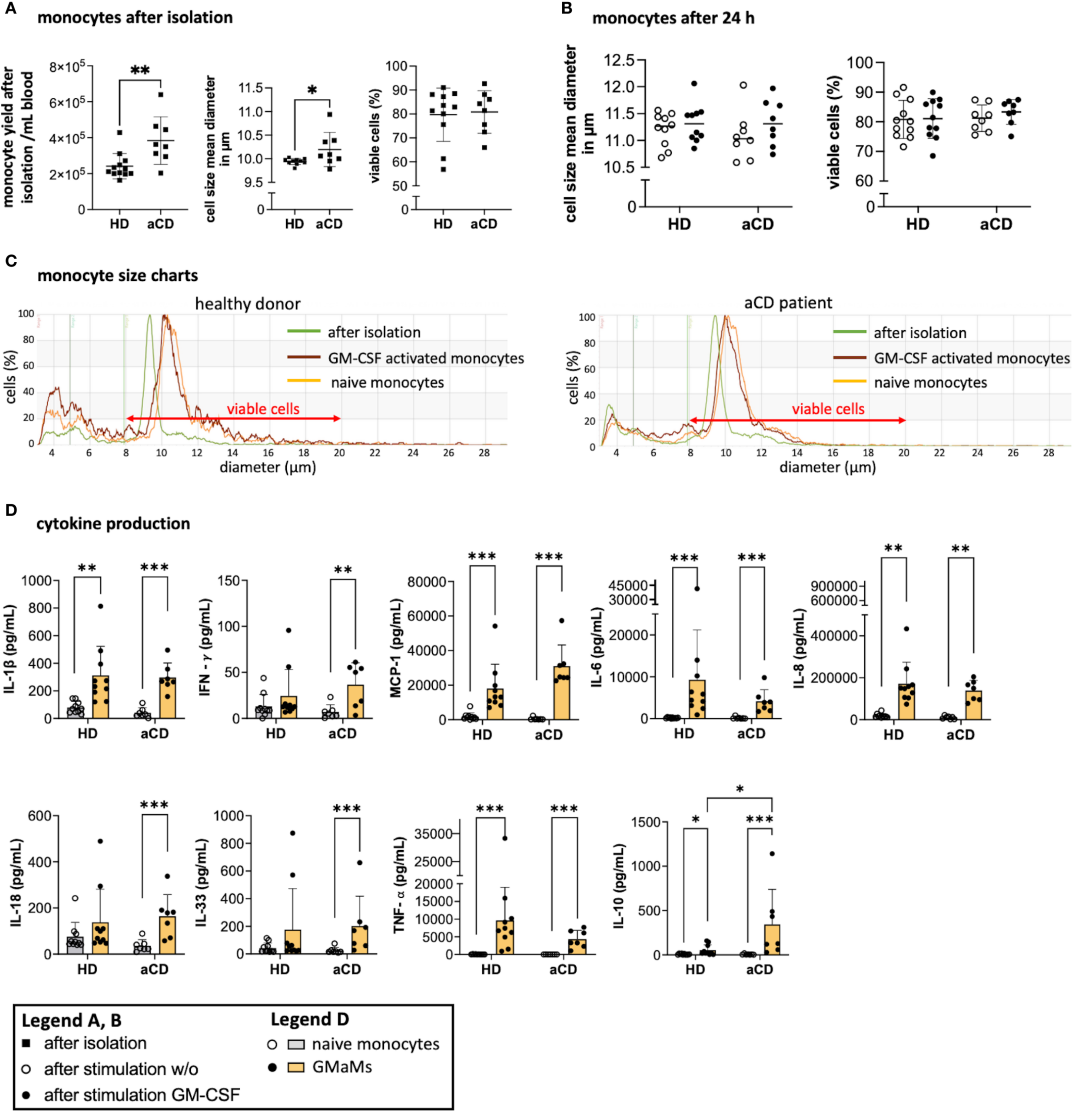
Monocyte properties after isolation and GM-CSF stimulation. Monocyte yield, size, and viability directly after isolation **(A)** and monocyte size and viability after 24h incubation with or without GM-CSF in suspension culture **(B)** were all measured using the CASY cell counter. A representative example of the gated monocyte population after isolation and incubation is shown based on cell diameter and cell counts depicting measurements after isolation (0h), and after incubation with or without GM-CSF (both at 44h) **(C)**. Cytokines were measured in cell culture supernatants after 24h of incubation with or without GM-CSF using LegendPlex^®^ or ELISA **(D)**. Results are presented as single dots for each individual and as bars indicating means ± SD. Pairwise comparisons were performed using the unpaired t-test **(A)** and Tukey procedure **(D)**. **(A)** n = 10, **(B–D)** n (HD) = 10, n(aCD) = 8; *p < 0.05, **p < 0.01, ***p < 0.001. Only significant comparisons are depicted. Abbreviations: aCD, active Crohn’s disease; GM-CSF, granulocyte-macrophage colony-stimulating factor; HD, healthy donors; IFN, interferon; IL, interleukin; MCP-1, monocyte chemoattractant protein 1; SD, standard deviation; TNF, tumor necrosis factor.

Immediately after monocyte isolation, yield, cell size, and viability were measured. Despite considerable variation in yield among individuals, patients with aCD had a higher number of peripheral monocytes compared to those with HD (**Figure 3A**). The cell size of monocytes from patients with aCD was significantly larger than that of monocytes from HDs (**Figure 3A**). No discernible difference in monocyte viability after isolation was observed (**Figure 3A**).

Following an *ex vivo* resting period and 24h GM-CSF activation (44h total), cell size was reassessed and no discernible difference was found between HDs and aCD patients regardless of GM-CSF activation (**Figure 3B**). Nevertheless, overall growth in cell size was observed throughout the culture period, as indicated by the shifted cell size peaks measured (**Figure 3C**). Moreover, GM-CSF activation led to increased secretion of several cytokines 24h post stimulation in both monocytes from HDs and aCD patients, including IL-1β, MCP-1, IL-6, IL-8, IL-18, TNF-α, and IL-10, as measured in the cell culture supernatants (**Figure 3D**). In contrast, significant increases in IFN-γ and IL-33 were observed only in samples from aCD patients (**Figure 3D**). Notably, IL-10 secretion was significantly higher in monocytes from aCD patients compared to those from HDs (**Figure 3D**), which was further reflected by an elevated TNF-α/IL-10 ratio in HD monocytes (**Supplementary Figure 3**).

In conclusion, monocytes from aCD patients exhibit a higher yield and size compared to those isolated from HDs, as well as an increased secretion of IL-10 following GM-CSF activation 24h after stimulation. Both groups exhibit elevated production of pro-inflammatory cytokines following GM-CSF activation; however, only aCD monocytes display significant increases in IFN-γ and IL-33.

### 3.2 Comparable functionality of GMaMs from patients with active CD and HDs

**Figure 4:**
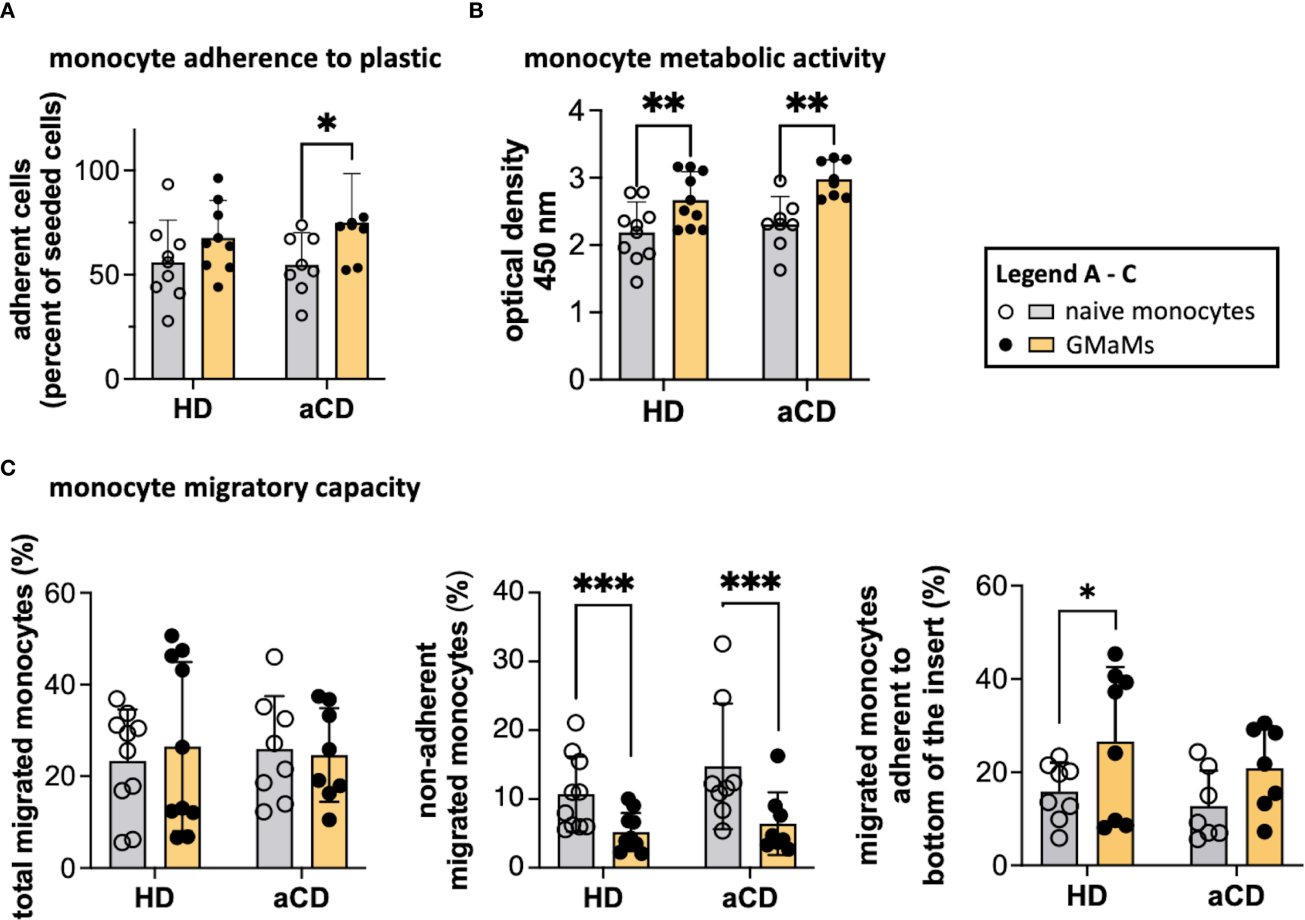
Functional tests of GMaMs and naïve monocytes. Adherence to the plastic surface was measured by counting adherent cells 2.5h after seeding onto a multi-well plate using a CASY cell counter **(A)**. Metabolic activity was assessed by measuring the optical density using a TECAN plate reader after incubation with WST-1 **(B)**. Migratory capacity was assessed by seeding monocytes in a transwell following an MCP-1 gradient and measuring the migrated monocytes after 4h using a CASY cell counter **(C)**. Results are presented as single dots for each individual and as bars indicating Means + SD. Multiple pairwise comparisons were performed using the Tukey procedure. **(A-C)** n(HD) = 8 – 10, n(aCD) = 7 – 8; *p < 0.05, **p < 0.01, ***p < 0.001. Only significant comparisons are depicted. aCD, active Crohn’s disease; GM-CSF, granulocyte-macrophage colony-stimulating factor; HD, healthy donors; MCP-1, monocyte chemoattractant protein 1; SD, standard deviation; WST-1, water-soluble tetrazolium salt.

After resting for 24h and undergoing GM-CSF stimulation, monocytes were harvested and subjected to functional tests, including adherence, metabolic activity, and migratory capacity. Naïve monocytes obtained from aCD patients did not exhibit higher adherence to plastic surfaces compared to monocytes obtained from HDs. However, GM-CSF activation resulted in increased adherence of monocytes from patients with aCD, whereas the increase in monocytes from HDs was not significant (**Figure 4A**). The metabolic activity of GMaMs, as measured by the WST assay, was increased in aCD patients and HDs compared to naïve cells (**Figure 4B**).

The total migratory capacity examination revealed no disparities between GMaMs and naïve monocytes (**Figure 4C**), regardless of whether the cells were from disease or control samples. However, when evaluating the percentage of monocytes adhering to the bottom of the transwell inserts after migration, a notable difference was observed. In HDs, there was an increased number of migrated GMaMs that adhered to the bottom of the transwell insert compared to naïve monocytes. In contrast, GM-CSF induced a significant decrease in the number of migrated non-adherent monocytes measured in the bottom chamber in samples from both patients and HDs (**Figure 4C**). In our experiments, the migration results were comparable regardless of whether MCP-1 was added as a chemoattractant; therefore, only the results with MCP-1 in the medium are shown in **Figure 4C**.

The increase in adherence and metabolic activity in monocytes from both aCD patients and healthy donors after GM-CSF stimulation was comparable, while overall migratory capacity remained unchanged. However, differences in post-migration adherence were observed between the groups.

### 3.3 aCD monocytes do not differ from HD monocytes in their protein surface expressions after GM-CSF activation

**Figure 5:**
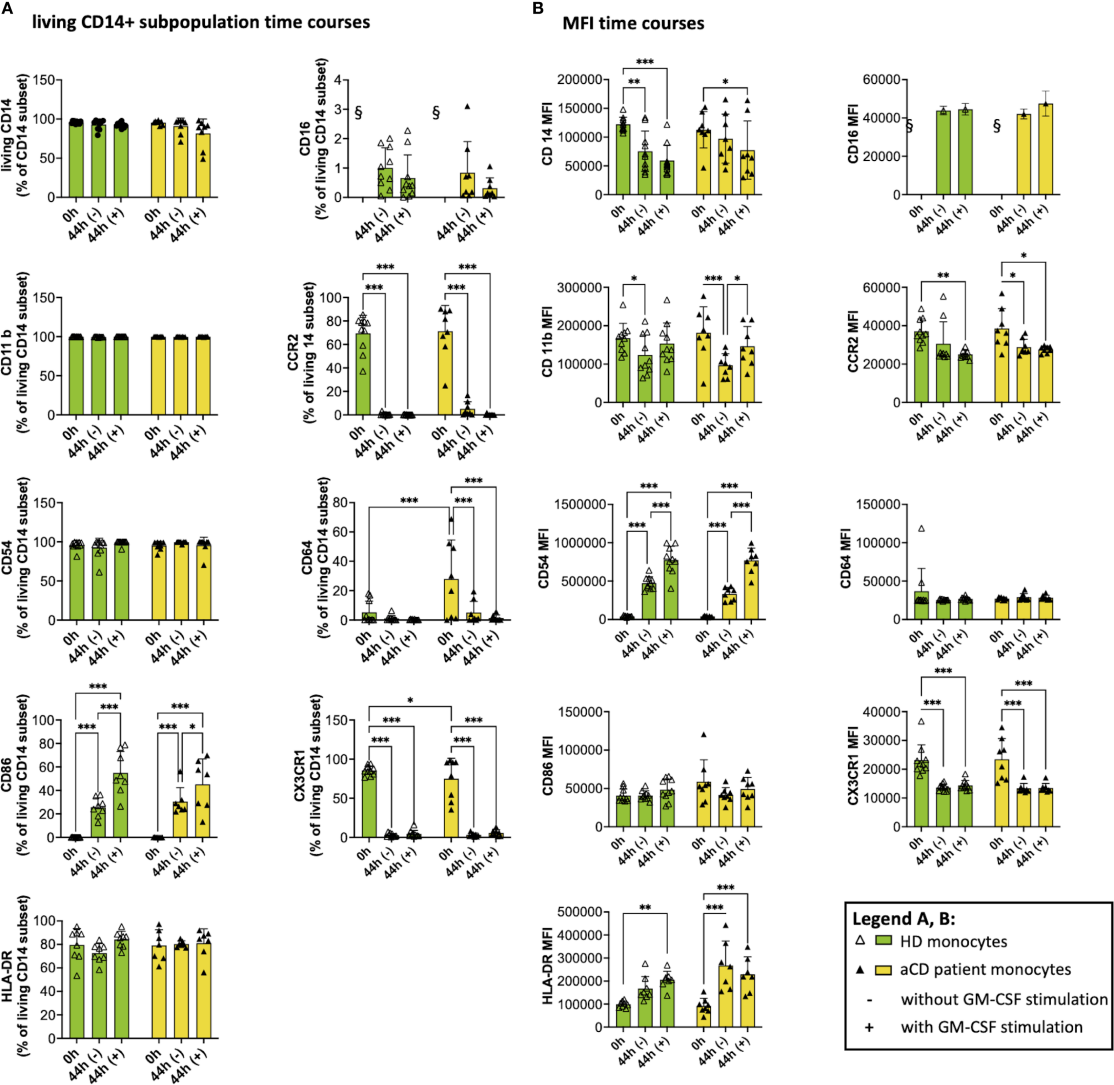
Surface marker expression and median fluorescence intensity (MFI) after isolation and after cell culture (with and w/o GM-CSF). The graphs show flow cytometry measurements of monocytes after isolation (0h) and after being incubated with (44h +) or without GM-CSF (44h -). A shows the subpopulation of living CD14^+^ monocytes expressing the respective surface marker, and B shows the MFI of monocytes. Both groups (healthy donors and patients with aCD) are shown. Surface marker thresholds were gated using fluorescence minus one (FMO). Results are presented as single dots for each individual and as bars indicating Means + SD. Multiple pairwise comparisons were performed using the Tukey procedure. **(A, B)** (HD) = 10, n(aCD) = 7 – 8; *p < 0.05, **p < 0.01, ***p < 0.001. § - no data because no CD16^+^ monocytes were isolated according to the selected isolation procedure. Only significant comparisons are shown. aCD, active Crohn’s disease; CD, cluster of differentiation; GM-CSF, granulocyte-macrophage colony-stimulating factor; GMaMs, GM-CSF-activated monocytes; HD, healthy donors; HLA, human leukocyte antigen; MFI, median fluorescence intensity; SD, standard deviation.

Monocytes were analyzed for expression of selected surface markers after isolation and after incubation with or without GM-CSF (**Figure 5**). Our data show that a consistently high percentage of CD14^+^ monocytes express CD11b, CD54, and HLA-DR on their surface (**Figure 5A**), irrespective of GM-CSF activation and disease status. Notably, directly after isolation (0 h), we observed a higher proportion of monocytes positive for CD64 in aCD compared to HDs, whereas the proportion of CX3CR1-positive monocytes was reduced in aCD monocytes compared to HD monocytes (**Figure 5A**).

While the proportions stayed comparable during cultivation, the median fluorescence intensity (MFI), indicating surface marker expression per cell, of CD11b decreased after cultivation in naïve monocytes of HDs. Notably, GMaMs derived from aCD patients expressed a significantly higher CD11b MFI than naïve monocytes in that group (**Figure 5B**). For CD54, an increase in MFI was observed after cultivation (44h), with a significantly higher increase in GMaMs compared to naïve monocytes, regardless of disease status (**Figure 5B**). The expression of the MCP-1 receptor CCR2 (CD192) decreased during monocyte cultivation (44h). In the aCD group, this decrease was significant in both GMaMs and naïve monocytes, whereas in the HD group, a significant reduction was observed only in GMaMs. Besides the decline of the MCP-1 receptor expression, GM-CSF activation also led to a comparable decrease in the proportion of cells expressing CCR2 (CD192) in aCD patients and HDs.

Fewer monocytes expressed CD64 after incubation with or without GM-CSF (44h), and while the difference was significant for aCD patients, it was not for HDs (**Figure 5A**). CD64 MFI stayed comparable in all groups.

The proportion of monocytes expressing CD86 was increased significantly after cultivation (44h), with a significantly higher increase in GMaMs in both the HD and aCD patient groups. Interestingly, no effect on the CD86^+^ MFI was detected (**Figures 5A, B**).

In most samples of GMaMs of both HD and aCD patients, less than 10% of monocytes were positive for the chemokine receptor CX3CR1, resulting in a significant decrease in this subpopulation (**Figure 5A**). Moreover, a significant decrease in the MFI of CX3CR1 was detected after culture in all groups (**Figure 5B**).

In summary, cultivation and GM-CSF activation altered the expression of multiple monocyte surface markers. Increases in CD54 MFI and decreases in CCR2 and CX3CR1 expression were observed in both HDs and aCD patients. Differences between groups included a significant increase in CD11b MFI only in GMaMs from aCD patients, a significant reduction of CD64 expression only in aCD patients, and elevated HLA-DR MFI in naïve monocytes exclusively in the aCD group. CD86 expression increased comparably in both groups without changes in MFI.

### 3.4 Cytokine response of GMaMs and naïve monocytes to LPS is comparable in HD and aCD monocytes except for IFN-γ and IFN-α2

To test how GMaMs respond to a potential bacterial stimulus, monocytes were stimulated with LPS for 24h, and inflammatory mediators were determined in supernatants, concurrent with the measurement of GMaMs’ metabolic activity.

**Figure 6:**
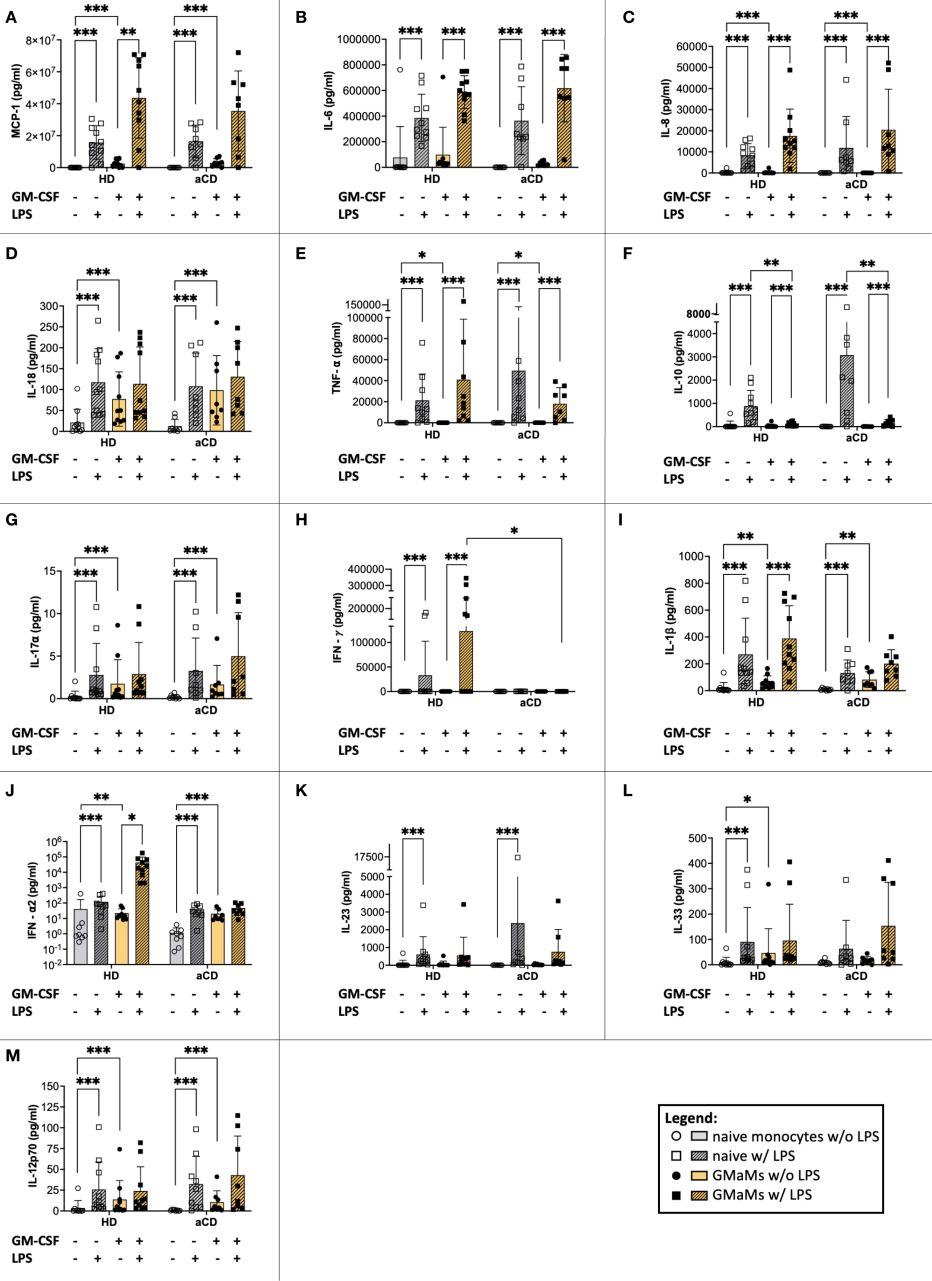
Response of GMaMs and naïve monocytes to LPS. Cytokine levels were measured after 24h of LPS stimulation using the LegendPlex™ or ELISA. Results are presented as single dots for each individual and bars indicate means + SD. Pairwise comparisons were performed using the Tukey test. **(J)** y-axis shown log 10 transformed to improve visualization. **(A-M)** n(HD) = 10, n(aCD) = 8; *p < 0.05, **p < 0.01, ***p < 0.001. Only significant comparisons are shown. aCD, active Crohn’s disease; ELISA, enzyme-linked immunoassay; GM-CSF, granulocyte-macrophage colony-stimulating factor; HD, healthy donors; IFN, interferon; IL, interleukin; LPS, lipopolysaccharides; MCP-1, monocyte chemoattractant protein 1; SD, standard deviation; TNF, tumor necrosis factor.

LPS stimulation resulted in increased cytokine secretion in both naïve monocytes and GMaMs across HDs and aCD patients (**Figures 6A–M**). MCP-1 (**Figure 6A**), IL-6 (**Figure 6B**), IL-8 (**Figure 6C**), IL-18 (**Figure 6D**), TNF-α (**Figure 6E**), IL-10 (**Figure 6F**), IL-1β (**Figure 6I**), IFN-α2 (**Figure 6J**), IL-12p70 (**Figure 6M**), and IL-17A (**Figure 6G**) levels were significantly elevated after LPS stimulation in naïve monocytes of HDs and aCD patients. GMaMs from HDs also showed increased secretion of these cytokines upon LPS treatment, with similar increases observed in GMaMs from aCD patients for IL-6 (**Figure 6B**), IL-8 (**Figure 6C**), TNF-α (**Figure 6E**), and IL-10 (**Figure 6F**). Additionally, GMaMs from both groups exhibited increased secretion of MCP-1 (**Figure 6A**), IL-8 (**Figure 6C**), IL-18 (**Figure 6D**), TNF-α (**Figure 6E**), IL-17A (**Figure 6G**), IL-1β (**Figure 6I**) and IL-12p70 (**Figure 6M**) compared to naïve monocytes in the absence of LPS. IL-10 secretion after LPS was higher in naïve monocytes compared to GMaMs in both groups (**Figure 6F**). The TNF-α/IL-10 ratio (**Supplementary Figure 3**) increased after LPS stimulation in both naïve and GM-CSF-activated monocytes, with higher values observed in GMaMs compared to naïve cells under both unstimulated and LPS-stimulated conditions in HDs and aCD patients. In addition, the IL-18/IL-10 ratio was increased in GMaMs from both HDs and aCD patients without LPS and was attenuated following LPS challenge. Naïve cells displayed a reduced IL-18/IL-10 ratio compared to GMaMs and showed no significant effect of LPS stimulation (**Supplementary Figure 3**). IFN-γ (**Figure 6H**) secretion increased after LPS in naïve and GMaMs of HDs but remained lower in GMaMs of aCD patients. For IFN-α2 (**Figure 6J**), higher levels were found in naïve and GMaMs of both groups after LPS, with GMaMs also showing increased secretion compared to naïve monocytes in the absence of stimulation. One discernible difference was the higher IFN-α2 secretion by HD GMaMs after LPS, which was not observed in aCD monocytes. IL-23 (**Figure 6K**) and IL-33 (**Figure 6L**) were elevated after LPS in naïve monocytes of HDs and aCD patients, with additional increased IL-33 secretion observed in GMaMs of HDs without LPS. IL-12p70 (**Figure 6M**) and IL-17A (**Figure 6G**) levels were also increased in GMaMs compared to naïve monocytes without stimulation.

In conclusion, LPS stimulation induced the secretion of multiple pro-inflammatory cytokines, including MCP-1, IL-6, IL-8, IL-18, TNF-α, IL-1β, IL-12p70, IL-17A, IL-23, and IFN-γ, in both naïve monocytes and GMaMs from HDs and aCD patients. Anti-inflammatory cytokines IL-10 and IFN-α2 were also increased in response to LPS. Additionally, GMaMs from both groups showed increased secretion of several pro-inflammatory cytokines compared to naïve monocytes in the absence of LPS. The TNF-α/IL-10 ratio increased following LPS stimulation and was higher in GMaMs compared to naïve monocytes under both stimulated and unstimulated conditions.

### 3.5 GM-CSF-induced changes are stable after 24h without further stimulation

Since LPS triggered a strong cytokine response in monocytes, the potential effects of GM-CSF during extended cell culture might be masked by the LPS-induced cytokine production. To address this, we separately analyzed unstimulated cells (naïve cells) to determine whether GM-CSF pretreatment led to changes in cytokine production after 24h without further stimulation by LPS.

**Figure 7:**
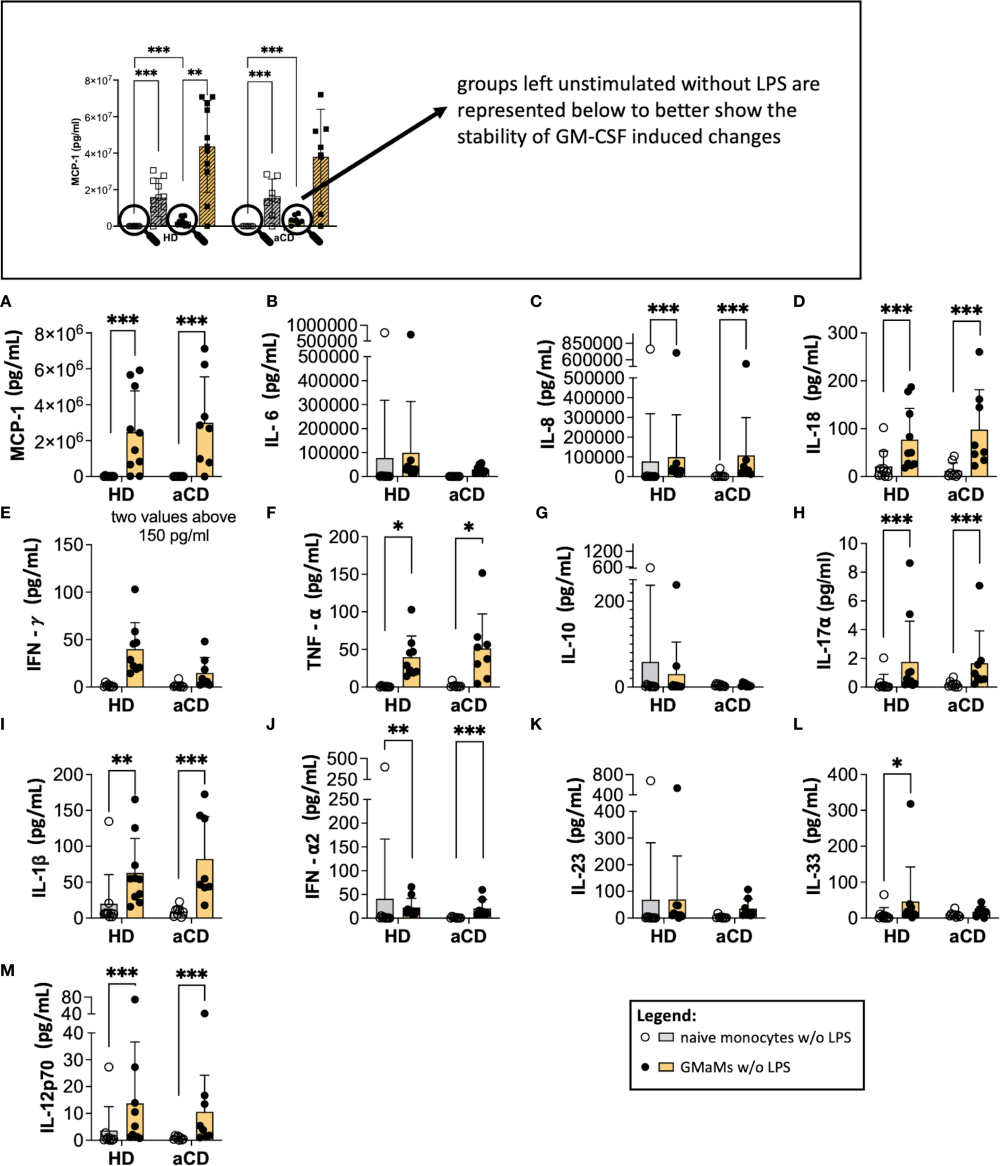
The phenotype of GMaMs after 24h without further stimulation. Cytokine levels were measured 24h after GM-CSF activation using the LegendPlex^®^ or ELISA. Bars indicate means + SD. Pairwise comparisons were performed using the Tukey Test. **(A-M)** n(HD) = 10, n(aCD) = 8; *p < 0.05, **p < 0.01, ***p < 0.001. aCD, active Crohn’s disease; GM-CSF, granulocyte-macrophage colony-stimulating factor; HD, healthy donors; IFN, interferon; IL, interleukin; LPS, lipopolysaccharide; MCP-1, monocyte chemoattractant protein 1; SD, standard deviation; TNF, tumor necrosis factor.

An increased cytokine secretion of MCP-1, IL-8, IL-18, TNF-α, IL1β, IFN-α2, IL-33, and IL-12p70 by GMaMs was detectable after 24h cultivation without LPS stimulation in both HDs and patients with aCD (**Figures 7A, C, D, F, I, J, L, M**). Furthermore, the TNF-α/IL-10 ratio of GMaMs was increased compared to naïve monocytes, regardless of disease status (**Supplementary Figure 3**).

These results indicate that, compared to naïve monocytes, the increased production of the cytokines mentioned above following GM-CSF activation is maintained in GMaMs for at least 24h. This included an elevated TNF-α/IL-10 ratio in GMaMs compared to naïve monocytes. This effect was comparable between HDs and aCD patients.

### 3.6 Attenuated KYN-pathway with increased QUIN and enhanced metabolic activity in GMaMs of aCD following LPS stimulation

**Figure 8:**
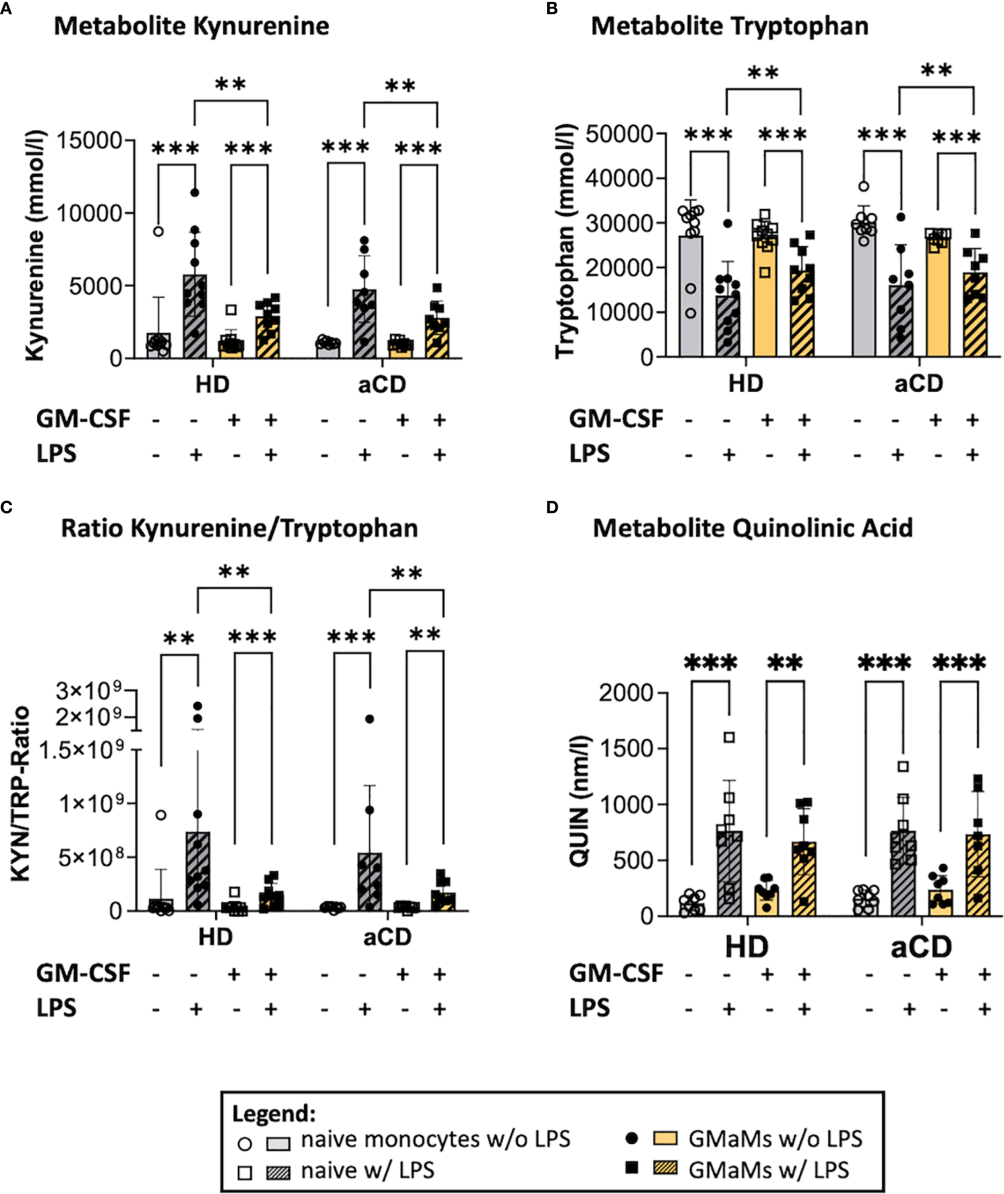
Metabolites and metabolic activity of GMaMs and naïve cells after incubation with or without LPS for 24h. Metabolites Kynurenin **(A)**, Tryptophan **(B)**, the Kynurenin/Tryptophan Ratio **(C)** as well as Quinolinic acid after 24h culture **(D)**. **(A-D)** n(HD) = 8, n(aCD) = 8; **p < 0.01, ***p < 0.001. aCD, active Crohn’s disease; GM-CSF, granulocyte-macrophage colony-stimulating factor; HD, healthy donors; KYN, Kynurenin; LPS, lipopolysaccharides; QUIN, quinolinic acid; SD, standard deviation; TRP, Tryptophan.

Following LPS treatment, both GMaMs and naïve monocytes exhibited a significant increase in KYN levels (**Figure 8A**), accompanied by a significant decrease in TRP levels (**Figure 8B**), regardless of disease status. However, in GMaMs, these LPS-induced changes were attenuated, resulting in a significantly lower increase of KYN and depletion of TRP in both monocytes from HD and aCD patients. Accordingly, a reduced KYN/TRP ratio was observed in GMaMs compared to naïve cells (**Figure 8C**). Additionally, we found a significant increase in QUIN levels in both GMaMs and naïve monocytes from both HD and aCD patients (**Figure 8D**). Furthermore, GMaMs of aCD patients displayed higher metabolic activity in the WST-1 assay after LPS treatment, whereas metabolic activity did not differ significantly between naïve monocytes and GMaMs of HDs (**Supplementary Figure 2**).

LPS-induced changes in TRP metabolism were attenuated in GMaMs compared to naïve monocytes. At the same time QUIN was elevated in all groups after LPS treatment. Additionally, GMaMs from aCD patients exhibited increased metabolic activity following LPS stimulation, compared to their naïve counterparts.

## 4 Discussion

This study investigated the *ex vivo* monocyte phenotype and physiology in patients with aCD and its implications for GMaMs as a potential therapeutic approach. Therefore, we aimed to examine whether the disease activity, potential nonspecific effects of medication, or intrinsic CD-associated impairments might modulate the phenotype of peripheral monocytes in patients with aCD. Our experiments focused on monocyte functions relevant to the intended application of GMaMs as a cell therapeutic, including migratory capacity, adherence, metabolic activity, surface markers, and cytokine response concurrent with pro- and anti-inflammatory responses to bacterial stimuli. Our findings demonstrate that GM-CSF stimulation induces comparable activation in monocytes of aCD patients and HDs, preserving key functional capacities relevant to potential therapeutic applications.

In detail, GM-CSF induced comparable activation in monocytes of both aCD patients and HDs characterized by increased metabolic activity, increased production of pro-inflammatory cytokines, and an enhanced capacity for adherence. The only exception was a GM-CSF-induced increase in anti-inflammatory IL-10 release, specifically in freshly isolated monocytes from aCD patients measured 24h post stimulation. This response was neither observed in healthy donors nor reported in monocytes from patients with quiescent CD (11). Accordingly, the TNF-α/IL-10 ratio was significantly lower in GMaMs of aCD patients, indicating a more balanced inflammatory response of monocytes after *in vitro* GM-CSF (21, 23). GM-CSF has been shown to induce IL-10 mRNA expression through Janus kinase 2 (JAK2) - signal transducer and activator of transcription 5 (STAT5) and extracellular signal-regulated kinase 1/2 (ERK1/2) pathways (35). Previous studies have shown that this induction has been transitory, with peak IL-10 mRNA levels occurring approximately 6h after stimulation (36), which may explain why the observed IL-10 increase is transient and was not detected in monocytes that were incubated for an additional 24h after stimulation. While our findings confirm a GM-CSF-induced transitory IL-10 increase in monocytic cell culture, previous studies also reported elevated IL-10 levels in the serum of patients with aCD (37) and demonstrated increased numbers of IL-10-producing mononuclear cells as well as higher IL-10 mRNA/protein abundance in inflamed mucosa (38), indicating active engagement of IL-10 induction *in vivo*. Interestingly, a GM-CSF–induced IL-10 response was observed in the monocytic cell line U937 (36), which might represent a chronically dysregulated inflammatory state due to tumor-promoting inflammation described in cancer cell lines (39). This supports the notion that chronic inflammation may prime peripheral monocytes for an exaggerated IL-10 response to GM-CSF. However, given the absence of comparable *in vivo* studies and the limited sample size of our cohort, these findings should be interpreted cautiously and warrant further investigation. Importantly, IL-10 is of interest as it can downregulate TNF-α production, contributing to the desirable effect of GMaMs in aCD patients, where a more controlled immune regulation is beneficial (40).

Irrespective of the differences in IL-10 response, GMaMs of aCD patients responded to LPS stimulation by excreting pro-inflammatory cytokines, such as MCP-1, IL-8, TNF-α, or IL-1β, comparable to the LPS responses observed in GMaMs of HDs. At the same time, a reduction in IL-10 secretion and a concurrent increase in the KYN/TRP ratio were observed in GMaMs from both aCD patients and HDs, reflecting a decreased anti-inflammatory mediator activity upon stimulation with LPS. This is further supported by our findings regarding the IL-18/IL-10 ratio. While IL-18 alone was increased by either GM-CSF or LPS regardless of disease status, when calculating the ratio to IL-10 we observed an increased ratio in GMaMs of both HD and aCD patients and an attenuated ratio by LPS challenge. Naïve cells expressed a reduced IL-18/IL-10 ratio compared to GMaMs and no significant LPS effect. These findings support our assumption that GMaMs are driven toward a pro-inflammatory state (24–26). In addition, we observed an increased QUIN production in the KYN/TRP pathway after LPS treatment, which indicates increased *de novo* NAD^+^ synthesis (29). NAD^+^ is required in the mitochondria for energy production through oxidative phosphorylation (OXPHOS), among other metabolites (41, 42). These results indicate an increased energy requirement of the LPS-challenged monocytes. Results of the monocytic cell line THP-1 revealed that challenge with bacterial LPS, a pro-inflammatory stimulus for macrophages, caused a large increase in NAD^+^ levels in pro- but not anti-inflammatory macrophages that were correlated with TNF-α release (43), supporting our findings of a pro-inflammatory response of GMaMs to LPS. These results are consistent with previous findings in naïve monocytes from CD patients in remission (11), suggesting that circulating monocytes from CD patients may share fundamental activation characteristics with those from healthy individuals, regardless of disease activity. Furthermore, these results are consistent with established theories suggesting GM-CSF promotes a pro-inflammatory monocyte phenotype (44). However, the *in vivo* function of myeloid effector cells and their respective activation endpoints are complexly regulated by the microenvironment in the inflamed tissue (45) and therefore difficult to estimate conclusively by our *ex vivo* experiments. Interestingly, we detected no secretion of IFN-γ after LPS stimulation in GMaMs of aCD, while GMaMs of HDs released IFN-γ. The impaired capacity for IFN-γ production was detected in patients with (n = 4) and without (n = 4) immunosuppressive medication (**Table 1**), which may indicate an intrinsic dysregulation of distinct innate immune response pathways to bacterial stimuli, irrespective of medication. These findings align with the perspective of CD as a form of innate immunodeficiency (12, 46), associated with dysfunctional macrophages, which are immune cells derived from monocytes. That dysfunction results in inadequate secretion of proinflammatory cytokines and chemokines upon bacterial challenge (13). Interestingly, in a study in mice, the therapeutic effect of GMaMs on colitis was accompanied by a decreased production of IFN-γ in the lamina propria by mononuclear cells *in vivo* (8). In this context, the diminished IFN-γ secretion observed in GMaMs from aCD patients may benefit their therapeutic effect by limiting excessive intestinal inflammation.

Our analyses indicate that monocytes from aCD patients exhibit further intrinsic signs of peripheral activation. This is supported by the higher proportion of cells positive for the surface marker CD64 in monocytes from aCD compared to HD directly after isolation (0h), as well as an increased yield and size of monocytes in aCD after isolation (47).

While the expression of CD64 is associated with chronic inflammatory diseases and promotes polarization to the proinflammatory M1 macrophage type (32, 33), increased production of IL-10 is associated with polarization in immunoregulatory macrophages (M2-like) (48). These results are in line with Däbritz et al. (8), describing that GMaMs represent an intermediate cell type that combines pro- and anti-inflammatory features in their early stage of development. Such a phenotype may be particularly advantageous in the inflammatory milieu of CD, where excessive immune activation coexists with impaired regulation. In this context, an influx of GMaMs may be beneficial despite their inflammatory potential, as CD is linked to defective GM-CSF signaling and insufficient innate clearance of microbes (7, 44–46). We suspect *ex vivo*–activated GMaMs may provide a focused, local recruitment of phagocytes by pro-inflammatory cytokines such as IL-8, MCP-1, and TNF-α. By trafficking to the inflamed gut tissue, GMaMs might restore deficient innate effector functions.

Importantly, Rahman et al. (12) pointed out that neutrophils provide the first cellular line of immune defense once epithelial barriers have been breached. *In vivo* and *ex vivo* models of neutrophil functions in CD showed that their reduced migration correlates with unusually low levels of chemotactic IL-8, mainly produced by intestinal macrophages (12). Our results show that *ex vivo* stimulation with GM-CSF induced a significant production of IL-8, and the resulting GMaMs exhibited an adequate and long-lasting response to LPS in terms of IL-8. Thus, GMaMs applied in aCD might compensate for impaired local chemotactic signaling, for example, IL-8 mediated, effectively recruiting neutrophils and monocytes to inflamed tissues despite intestinal macrophage dysfunction. Moreover, the significant secretion of MCP-1 by GMaMs in response to LPS, as shown in our experiments, could enhance endogenous monocyte recruitment to the site of inflammation, potentially enhancing the therapeutic effect. Whether the properties shown are therapeutically effective cannot be conclusively clarified *ex vivo*, as intestinal inflammation is too complex to be depicted *ex vivo*.

Our analyses of the migratory capacity revealed that GMaMs were able to migrate through a transwell membrane but adhered to the bottom of the transwell insert, while more naïve cells accumulated in the lower chamber of the transwell assay without adherence to the insert. The increased capacity for adherence of GMaMs was further supported by an increased expression of the surface marker CD54 (ICAM-1), known to mediate adhesion and trans-endothelial migration (49). Additionally, GM-CSF activation prevented the culture-induced reduction of CD11b, which is linked to the adhesion of monocytes to the endothelium in the early inflammatory response (50). Although GMaMs effectively migrated through the transwell membrane, the addition of the chemoattractant MCP-1 did not further enhance migration, possibly due to the observed time-dependent downregulation of its receptor, CCR2 (CD192) (51). This process was accompanied by a reduction in monocytes positive for the surface protein CX3CR1, a receptor for the chemoattractant fractalkine (CX3CL1), which promotes tight, integrin-independent adhesion of monocytes. It is expressed by several cell types, e.g., activated vascular endothelial cells, epithelial cells, dendritic cells or macrophages (52). The observed reduction in these two chemoattractant receptors may reflect the transitional state of peripheral classical monocytes after their release from the bone marrow. In circulation, classical monocytes either differentiate into intermediate monocytes (1% of classical monocytes) or undergo death or migration after a circulating lifespan of ∼approximately 1 day (53). Our experiments demonstrated a high proportion of viable cells after 44h of cultivation, regardless of GM-CSF activation. The absence of cell death, combined with the increasing size of monocytes, suggests that *ex vivo* culture is associated with the remodeling of surface molecules related to the early stages of monocyte differentiation. This was supported by the increase of markers relevant to cell-cell interaction, such as HLA-DR, which is an antigen-presenting T-cell receptor essential for the activation of the adaptive immune response (54) and CD86, a co-stimulatory molecule expressed on monocytes and (together with CD80) essential for activating lymphocytes and thus adaptive immunity (55). Both were elevated in GMaMs from aCD patients and HDs. Our results show that HLA-DR surface expression increased significantly in both naïve and GM-CSF-treated monocytes from aCD patients during culture. In contrast, monocytes from HDs showed a significant increase only after GM-CSF activation. This suggests that monocytes from aCD patients already exhibited elevated HLA-DR expression at baseline, likely due to endogenous inflammatory stimulation. This assumption is supported by the increased CD64 expression, yield, and size of monocytes of aCD patients, observed directly after isolation. However, this stimulation, which might have been induced by the active disease status, did not affect the culture- and GM-CSF-induced remodeling of monocyte surface markers, which was comparable between monocytes from aCD and HD. Whether the internalization of receptors CX3CR1 and CCR2 (CD192) potentially impairs the recruitment of GMaMs and, thus, the therapeutic benefit cannot be conclusively clarified in this study. It is well-established that monocyte migration in IBD is a complex process that extends beyond a singular pathway focus and is mediated by a multitude of chemotactic and adhesive mediators at the sites of inflammation, as well as blood components and the intestinal environment (56). Furthermore, it remains unclear if the GMaM phenotype is associated with a monocyte-derived dendritic cell-like or macrophage-like phenotype, which should be addressed in further studies focusing on specific markers for each cell type. In our study, spill-over was observed for some surface markers in flow cytometry, which represents a limitation for analyzing certain monocyte populations. Accordingly, for ongoing studies on surface markers of GMaMs, the staining protocol should be optimized to improve the resolution of specific subsets.

In conclusion, our findings support our hypothesis that, despite active inflammation, peripheral monocytes from aCD patients retain their functional responsiveness to GM-CSF activation, albeit within the limitations of small sample size (n = 8). The GMaM phenotype was characterized by increased metabolic activity, enhanced production of inflammatory cytokines, increased adhesion, and the remodeling of cell surface receptors towards molecules relevant for T-cell activation. We could affirm that monocytes from aCD patients, like monocytes from CD patients in remission, were not impaired in their functionality regarding migration, adhesion, and cytokine response to LPS stimulation (11). The response of GMaMs to LPS was comparable between aCD patients and HDs and included an increased production of pro-inflammatory and chemotactic cytokines such as IL-8 and MCP-1, which are crucial for the recruitment of monocytes or neutrophils to the site of inflammation. This mechanism might compensate for a previously hypothesized innate immune deficiency in CD (7, 57–59). The reduced ratio of TNF-α to IL-10 revealed a more balanced inflammatory response in GMaMs, potentially contributing to the desirable effect of GMaMs in aCD. This might be advantageous in CD, where excessive TNF-α contributes to pathology. The diminished LPS-induced IFN-γ production displayed by GMaMs of aCD patients may reflect a potential intrinsic dysregulation of innate immune responses. However, there is no indication that the reduced release of IFN-γ represents a limitation for the intended therapeutic use. Additionally, our findings suggest that, while GMaMs sustain the ability to migrate and adhere, the reduction of certain receptors may affect their *in vivo* recruitment and therefore warrant further investigation to determine the impact on their therapeutic potential.

## Data Availability

The raw data supporting the conclusions of this article will be made available by the authors, without undue reservation.

## Glossary

aCD: active Crohn’s disease
ADCC: antibody-dependent cellular cytotoxicity
ANOVA: analysis of variance
APC: allophycocyanin
ATP: adenosine triphosphate
CDAI: Crohn’s Disease Activity Index
aCD: active Crohn’s disease
CCR2 (CD192): chemokine (C-C motif) receptor 2
CCL2: C-C motif ligand 2
CE: collision energy
CO₂: carbon dioxide
CRP: C-reactive protein
CTLA-4: cytotoxic T-lymphocyte-associated protein 4
CV: coefficient of variation
CX3CR1: chemokine (C-X3-C motif) receptor 1
DP: declustering potential
DMSO: dimethyl sulfoxide
EDTA: ethylenediaminetetraacetic acid
ELISA: enzyme-linked immunosorbent assay
FBS: fetal bovine serum
FcγRI: Fc gamma receptor I
FITC: fluorescein isothiocyanate
FMO: fluorescence minus one
FSC-A: forward scatter area
GI: gastrointestinal
GM-CSF: granulocyte-macrophage colony-stimulating factor
GMaMs: GM-CSF-activated monocytes
Hb: hemoglobin
HD: healthy donors
HLA-DR: human leukocyte antigen-DR
ICAM-1: intercellular adhesion molecule 1
IDO: Indoleamine 2,3-dioxygenase
IFN: interferon
IgG: immunoglobulin G
IL: interleukin
KYN: kynurenine
LC-MS/MS: liquid chromatography-tandem mass spectrometry
LPS: lipopolysaccharide
MCP-1: monocyte chemoattractant protein 1
MFI: median fluorescence intensity
MHC: major histocompatibility complex
NIR: near-infrared
n/a: not available
NAD⁺: nicotinamide adenine dinucleotide
OXPHOS: oxidative phosphorylation
PBS: phosphate-buffered saline
PE: phycoerythrin
PerCP: peridinin-chlorophyll-protein complex
QC: quality control
QUIN: quinolinic acid
RPMI: Roswell Park Memorial Institute medium
SD: standard deviation
SSC-A: sideward scatter area
TLR: toll-like receptor
TNF: tumor necrosis factor
TRP: tryptophan
w/o: without
WST-1: water-soluble tetrazolium salt
