# Supplementary material for: Peripheral monocytes from Crohn’s disease patients retain functional responsiveness to GM-CSF during active disease
Source: Front Immunol. 2025 Sep 23;16:1663713. doi: 10.3389/fimmu.2025.1663713 (PMC12500572; doi:10.3389/fimmu.2025.1663713)
Supplement: Supplementary file 1 [file DataSheet1.pdf]

Supplementary Material

1 Supplementary Figures and Tables

1.1 Supplementary Figures

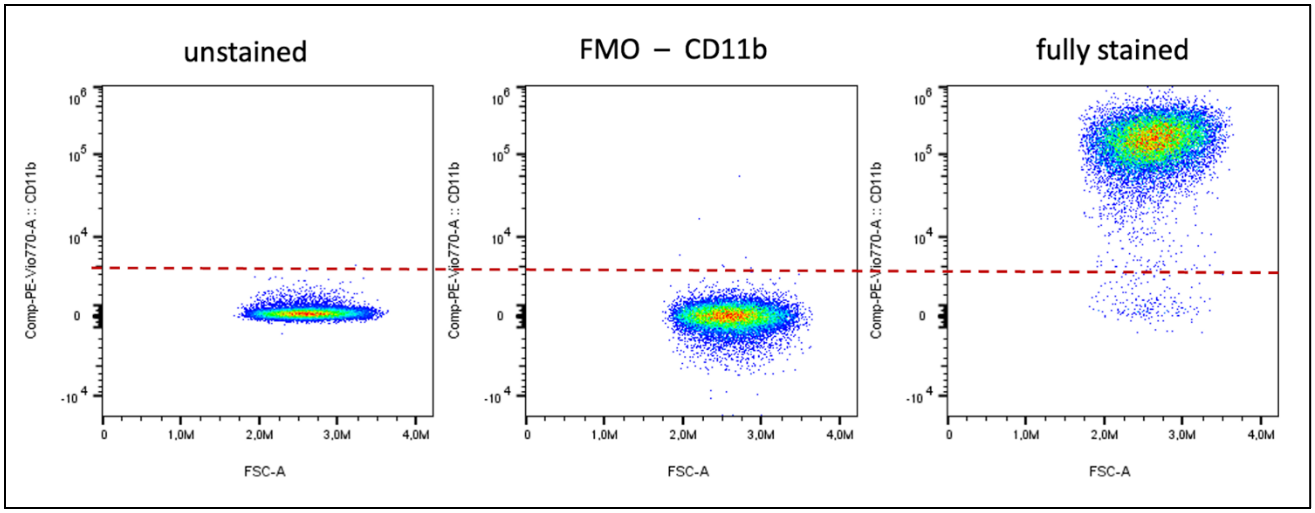

Supplementary Figure 1. Fluorescence minus one (FMO) control to determine fluorescence spread. Multicolor dot plots are reflecting the fluorescence spread into the PE-Vio770 channel (used for CD11b) shown by the FMO control compared to an unstained control. The red dotted line represents FMO gating boundary compared to the unstained boundary and is used as a threshold for the respective surface marker in a cell population. Abbreviations: CD, cluster of differentiation; FMO, Fluorescence minus one; PE, phycoerythrin.

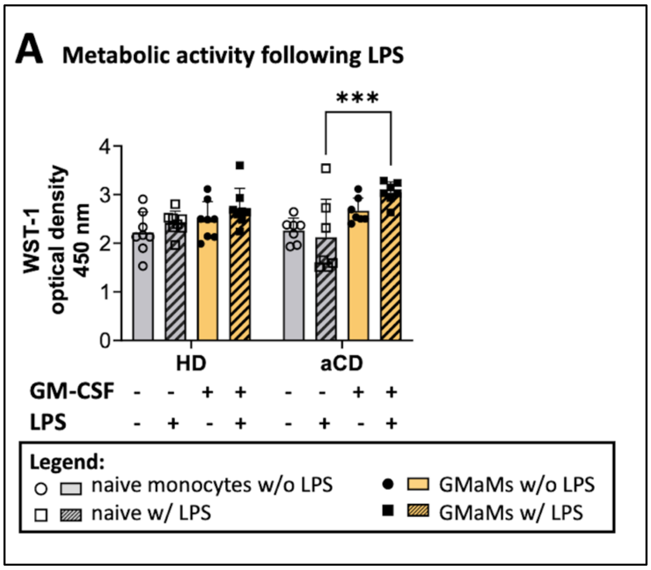

Supplementary Figure 2. Metabolic activity was assessed using the WST-1 assay after 24 h of LPS stimulation by measuring the optical density using a TECAN plate reader after incubation with WST-1. Results are presented as single dots for each individual and bars indicate means + SD. Pairwise comparisons were performed using the Tukey test. Abbreviations: aCD, active Crohn's disease; HD, healthy donors; LPS, lipopolysaccharides; SD, standard deviation; WST-1, water-soluble tetrazolium salt.

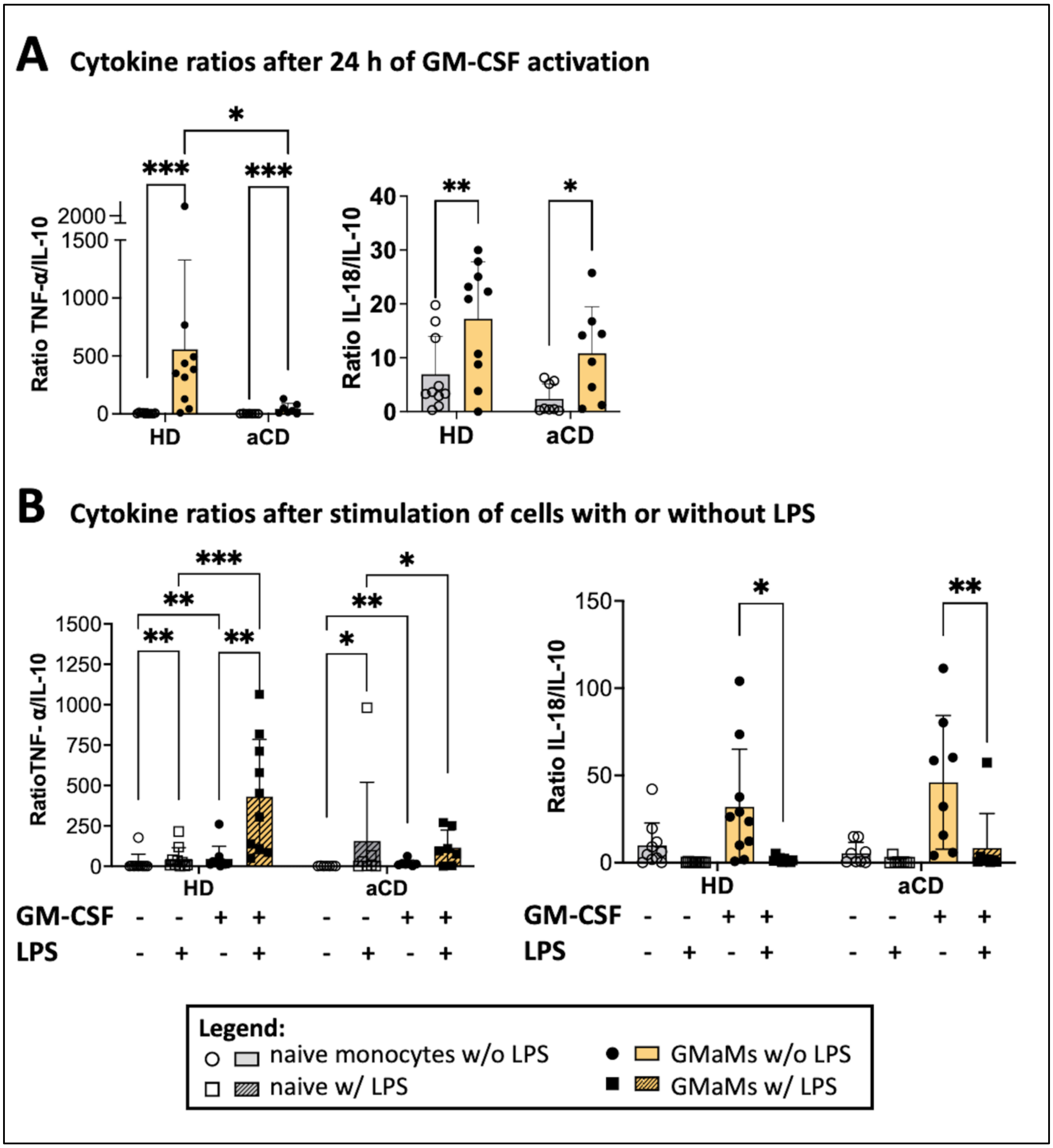

Supplementary Figure 3. Cytokine ratios of cytokines measured 24 h after GM-CSF activation (**A**) and after 24 h of stimulation of activated or naïve monocytes with or without LPS were calculated. Cytokines were measured using the LegendPlex™. Results are presented as single dots for each individual and bars indicate means + SD. Pairwise comparisons were performed using the Tukey test. (**A, B**): n(HD) = 10, n(aCD) = 8; * $p < 0.05$, ** $p < 0.01$, *** $p < 0.001$. Only significant comparisons are shown. Abbreviations: aCD, active Crohn's disease; GM-CSF, granulocyte-macrophage colony-stimulating factor; HD, healthy donors; IL, interleukin; LPS, lipopolysaccharides; SD, standard deviation; TNF, tumor necrosis factor.

Supplementary Tables

Supplementary Table 1. Mass transitions, declustering potential (DP), and collision energy (CE) for Tryptophan and Kynurenine and their internal standards in the method used.

Analyte	Q1 (m/z)	Q3 (m/z)	DP (V)	CE (V)
**Tryptophan**	205,100	118,000	39	23
**Kynurenine**	209	94	41	20
**Quinolinic acid**	168	124	46	17
**D6-Kynurenine**	215	198	61	15
**D5-Tryptophan**	210	122	31	37
**D5-Phenylalanin**	171	125	66	21

Abbreviations: CE, collision energy; DP, declustering potential.

Supplementary Table 2: Within- and between-run precisions for determination of tryptophan and kynurenine at different concentrations (low/mid/high). Precision is expressed as the coefficient of variation (CV).

Analyte	Analytical range (nmol/L)	Precision	QC low CV (%)	QC mid CV (%)	QC high CV (%)
**Tryptophan**#	100 – 204,800	Within-run	0.6 – 2.4	0.8 – 2.3	2.6 – 5.6
		Between-run	5.4 – 5.8	3.1 – 5.9	6.6 – 7.5
**Kynurenine**#	10 – 20,480	Within-run	0.9 – 3.3	0.8 – 3.8	3.4 – 7.6
		Between run	12.2 – 12.4	7.8 – 8.17	7.6 – 7.9
**Quinolinic acid**$	40 – 5,120	Within-run	15.6	N.A.	8.6
		Between-run	29.4	N.A.	17.8

#QC low: Medium (RPMI1640 + 10% fetal bovine serum); QC mid: Medium + 5 µmol/L of tryptophan and kynurenine; QC high: Medium + 50 µmol/l of tryptophan and kynurenine. To calculate the within-run precision, the QCs were measured in six replicates on three different plates on different days. In the table, the observed range of precision is given. To estimate the between-run precision, three samples of QC low, mid, and high were measured on six different plates. In the table, the observed range of precision of three samples is shown. $ The QC low was prepared by pooling human serum (20 healthy donors) with 10 µL of internal standard mix and measured with six replicates for calculation of within—and between-run precision. QC high was prepared of QC 1 spiked with 500 nM of quinolinic acid. Abbreviations: CV, coefficient of variation; QC, quality control; N.A., data not available.

Supplementary Table 3: Surface markers used in our flow cytometry panel, their function and references.

Surface Marker	Physiological function	Fluorochrome used	References
**CD14**	LPS receptor as co-receptor with toll-like receptors (TLRs); marker for monocytes and macrophages	PerCP Vio 700	(22, 42)
**CD16**	Marker for Antibody-Dependent Cellular Cytotoxicity (ADCC); monocyte marker	VioBlue	(43)
**CD11b (integrin alpha M)**	Receptor for immune cell interaction and migration; receptor for enhancement of immunological response (e.g., TNF-alpha expression)	PE-Vio 770	(44, 45)
**CX3CR1**	Receptor for mediating chemotaxis, adhesion, and survival of monocytes in response to its ligand CX3CL1 (fractalkine)	PE	(46)
**CCR2 (CD192)**	MCP-1 (CCL2) receptor	Vio-Bright FITC	(35)
**CD54 (ICAM-1)**	Cell adhesion molecule (ICAM-1); stimulating T-cell response	APC	(33, 47)
**CD64 (FcγRI)**	Mediation of immune responses in inflammation, including phagocytosis, ADCC, and the release of pro-inflammatory cytokines by binding to the Fc region of immunoglobulin G (IgG)	PE Vio 615	(48, 49)
**CD86**	Co-stimulatory molecule, provides secondary signals for T-cell activation and survival by binding to CD28 and CTLA-4 on T cells	Super Bright 645	(39)

**HLA-DR**	A major histocompatibility complex (MHC) class II receptor presenting extracellularly derived peptides to CD4+ T cells	VioGreen	(38)
**Zombie NIR**	Cell vitality (identifying necrotic cells)	NIR	(50)

Abbreviations: ADCC, Antibody-Dependent Cellular Cytotoxicity; APC, Allophycocyanin; CCL2, C-C motif ligand 2; CD, cluster of differentiation; CTLA-1, Cytotoxic T-lymphocyte-associated protein 4; FITC, Fluorescein isothiocyanate; ICAM-1, intercellular adhesion molecule 1; IgG, Immunoglobulin G; LPS, Lipopolysaccharide; MCP-1, Monocyte chemoattractant protein-1; NIR, near-infrared; PE, phycoerythrin; PerCP, peridinin-chlorophyll-protein complex; TLR, toll-like receptor.
